# Retraction Note: Involvement of autophagy in hypoxia-BNIP3 signaling to promote epidermal keratinocyte migration

**DOI:** 10.1038/s41419-024-06709-3

**Published:** 2024-05-06

**Authors:** Junhui Zhang, Can Zhang, Xupin Jiang, Lingfei Li, Dongxia Zhang, Di Tang, Tiantian Yan, Qiong Zhang, Hongping Yuan, Jiezhi Jia, Jiongyu Hu, Jiaping Zhang, Yuesheng Huang

**Affiliations:** 1 Institute of Burn Research, State Key Laboratory of Trauma, Burns and Combined Injury, Southwest Hospital, Army Medical University (Third Military Medical University), Chongqing, China; 2 Military Burn Center, The 990th (159th) Hospital of People’s Liberation Army, Zhumadian, China; 3 Endocrinology Department, Southwest Hospital, Army Medical University (Third Military Medical University), Chongqing, China; 4 Department of Plastic Surgery, Southwest Hospital, Army Medical University (Third Military Medical University), Chongqing, China

Retraction to: *Cell Death and Disease* 10.1038/s41419-019-1473-9, published online 08 March 2019

The Editors-in-Chief have retracted this article because of concerns about two of the figures:

- The BPI3 band in Figure 3A is repeated in Figure 5C
- The panels for Norm+shNC and Hypo+shBNIP3 in Figure 3E partially overlap

Junhui Zhang disagrees with this retraction. Can Zhang, Xupin Jiang, Lingfei Li, Dongxia Zhang, Di Tang, Tiantian Yan, Qiong Zhang, Hongping Yuan, Jiezhi Jia, Jiongyu Hu, Jiaping Zhang and Yuesheng Huang have not responded to correspondence from the Publisher about this retraction.

